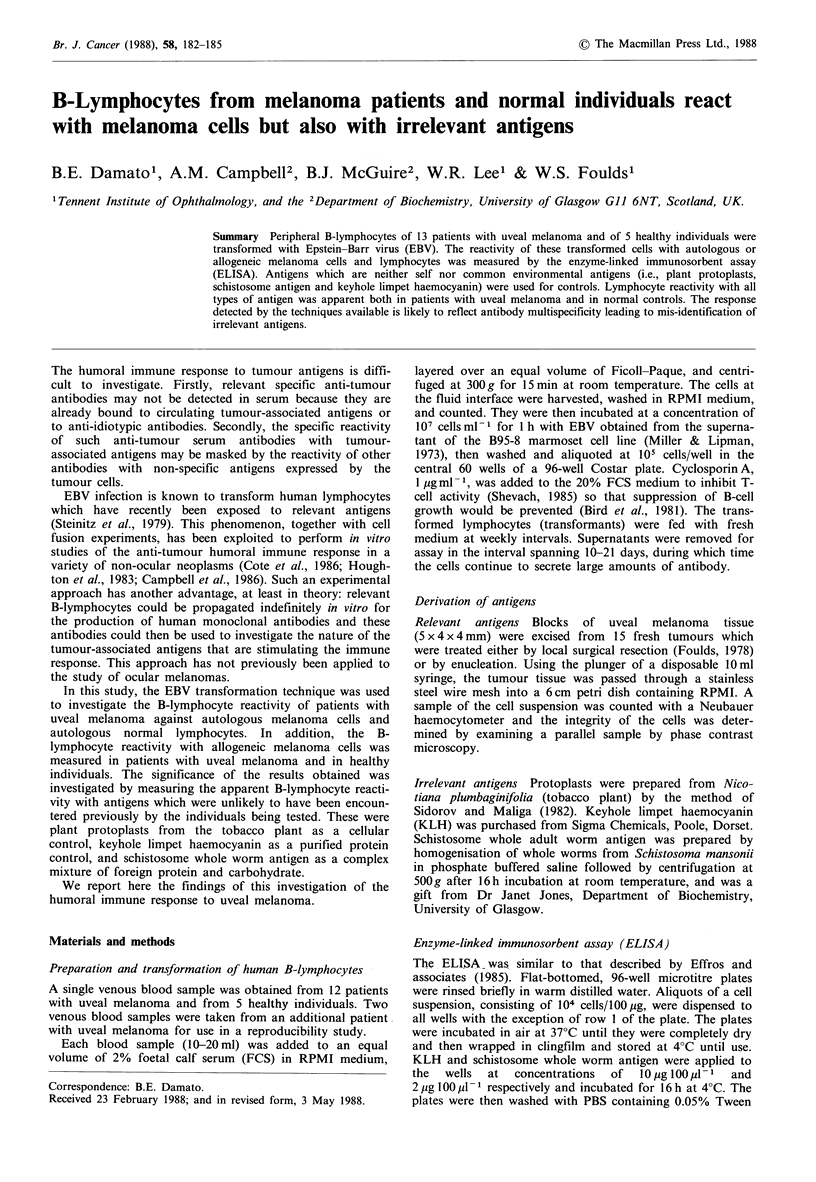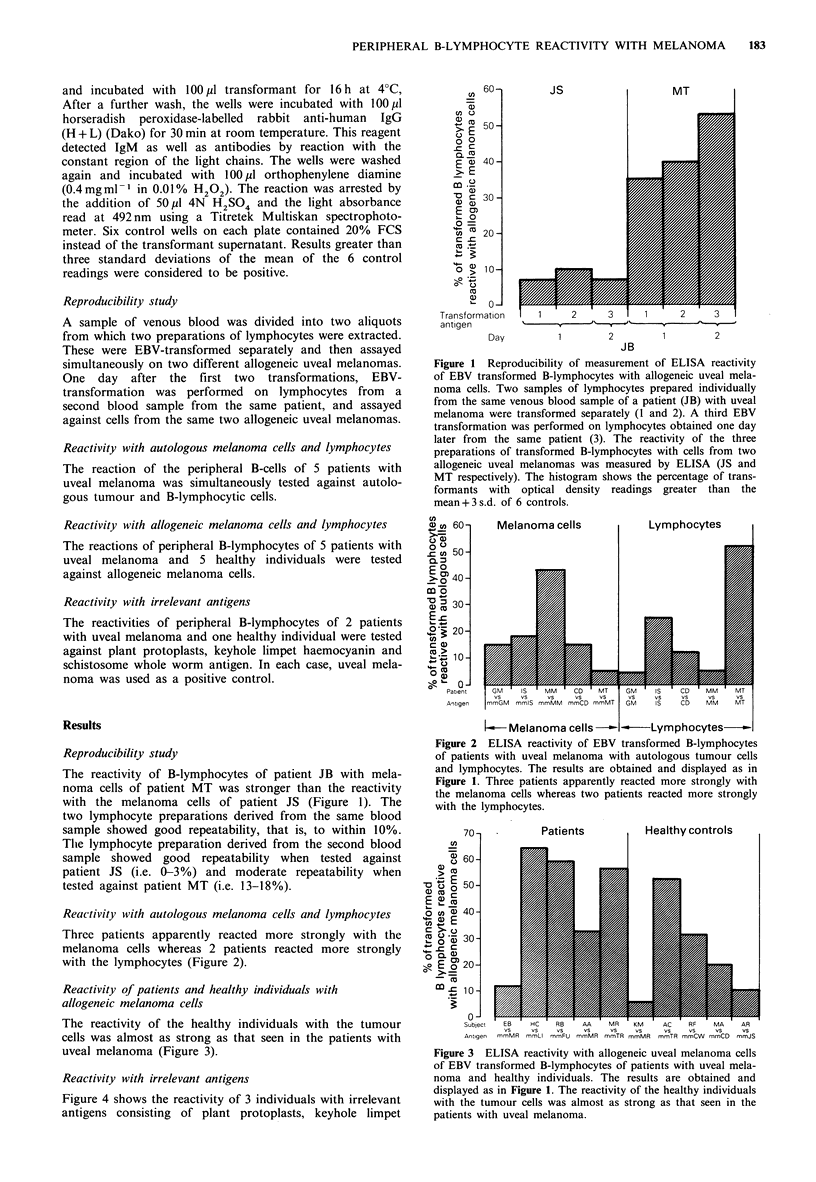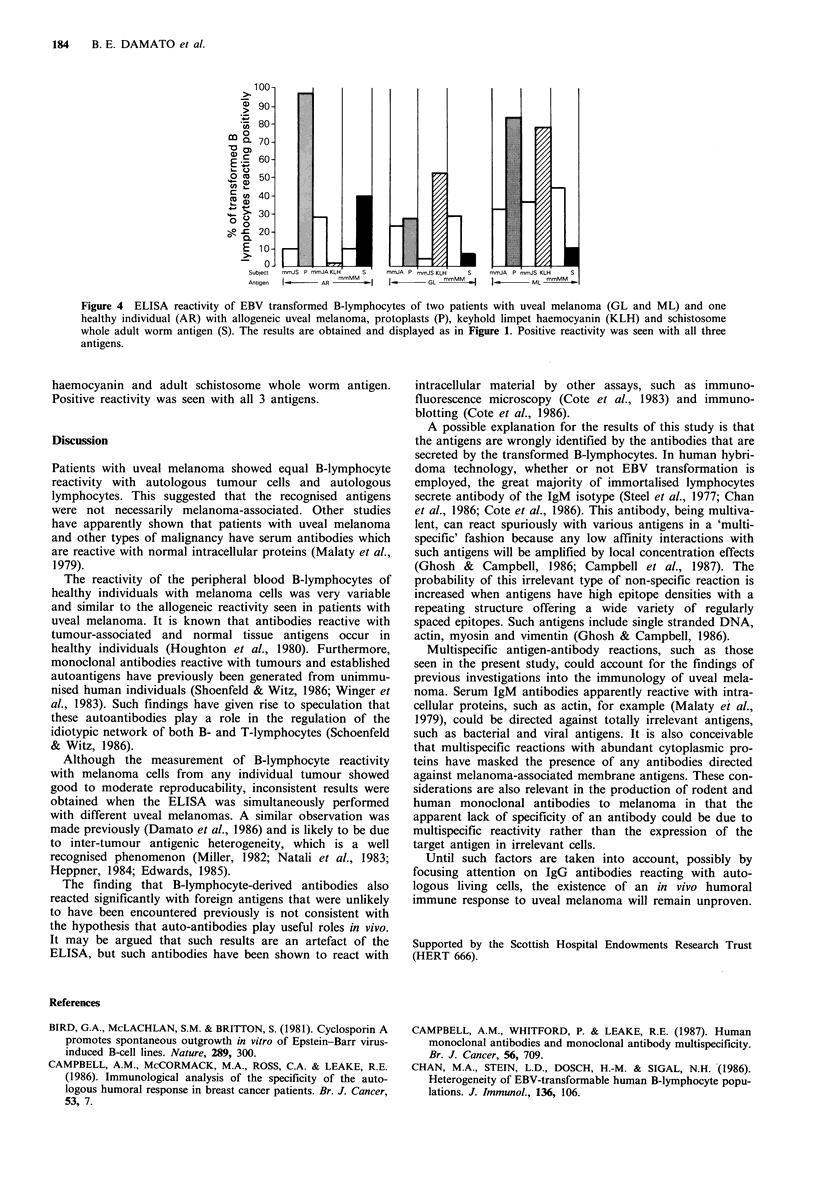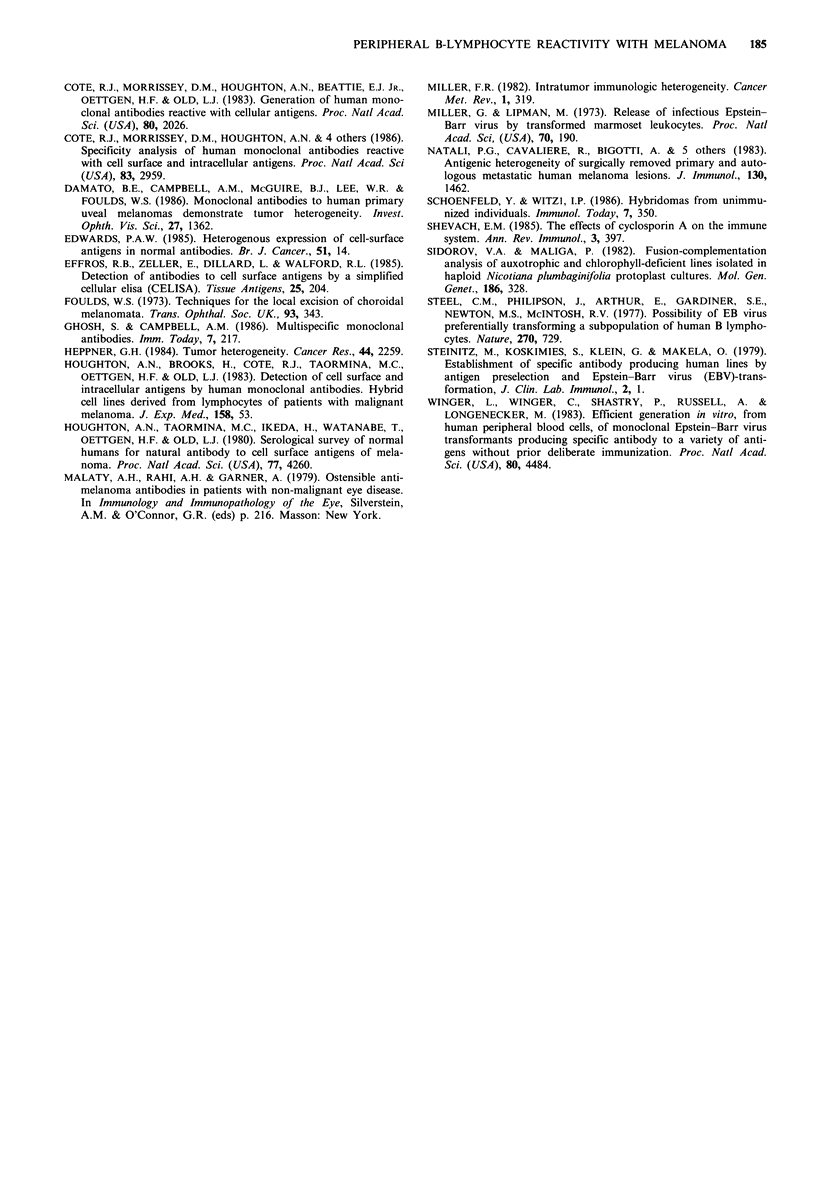# B-lymphocytes from melanoma patients and normal individuals react with melanoma cells but also with irrelevant antigens.

**DOI:** 10.1038/bjc.1988.188

**Published:** 1988-08

**Authors:** B. E. Damato, A. M. Campbell, B. J. McGuire, W. R. Lee, W. S. Foulds

**Affiliations:** Tennent Institute of Ophthalmology, University of Glasgow, Scotland, UK.

## Abstract

Peripheral B-lymphocytes of 13 patients with uveal melanoma and of 5 healthy individuals were transformed with Epstein-Barr virus (EBV). The reactivity of these transformed cells with autologous or allogeneic melanoma cells and lymphocytes was measured by the enzyme-linked immunosorbent assay (ELISA). Antigens which are neither self nor common environmental antigens (i.e., plant protoplasts, schistosome antigen and keyhole limpet haemocyanin) were used for controls. Lymphocyte reactivity with all types of antigen was apparent both in patients with uveal melanoma and in normal controls. The response detected by the techniques available is likely to reflect antibody multispecificity leading to mis-identification of irrelevant antigens.


Br. J. Cancer (1988), 58, 182–185 © The Macmillan Press Ltd., 1988

### B-Lymphocytes from melanoma patients and normal individuals react with melanoma cells but also with irrelevant antigens

B.E. Damato[1], A.M. Campbell[2], B.J. McGuire[2], W.R. Lee[1] & W.S. Foulds[1]

[1]Tennent Institute of Ophthalmology, and the [2]Department of Biochemistry, University of Glasgow G11 6NT, Scotland, UK.

**Summary** Peripheral B-lymphocytes of 13 patients with uveal melanoma and of 5 healthy individuals were transformed with Epstein–Barr virus (EBV). The reactivity of these transformed cells with autologous or allogeneic melanoma cells and lymphocytes was measured by the enzyme-linked immunosorbent assay (ELISA). Antigens which are neither self nor common environmental antigens (i.e., plant protoplasts, schistosome antigen and keyhole limpet haemocyanin) were used for controls. Lymphocyte reactivity with all types of antigen was apparent both in patients with uveal melanoma and in normal controls. The response detected by the techniques available is likely to reflect antibody multispecificity leading to mis-identification of irrelevant antigens.

The humoral immune response to tumour antigens is difficult to investigate. Firstly, relevant specific anti-tumour antibodies may not be detected in serum because they are already bound to circulating tumour-associated antigens or to anti-idiotypic antibodies. Secondly, the specific reactivity of such anti-tumour serum antibodies with tumour-associated antigens may be masked by the reactivity of other antibodies with non-specific antigens expressed by the tumour cells.

EBV infection is known to transform human lymphocytes which have recently been exposed to relevant antigens (Steinitz *et al*., 1979). This phenomenon, together with cell fusion experiments, has been exploited to perform *in vitro* studies of the anti-tumour humoral immune response in a variety of non-ocular neoplasms (Cote *et al*., 1986; Houghton *et al*., 1983; Campbell *et al*., 1986). Such an experimental approach has another advantage, at least in theory: relevant B-lymphocytes could be propagated indefinitely *in vitro* for the production of human monoclonal antibodies and these antibodies could then be used to investigate the nature of the tumour-associated antigens that are stimulating the immune response. This approach has not previously been applied to the study of ocular melanomas.

In this study, the EBV transformation technique was used to investigate the B-lymphocyte reactivity of patients with uveal melanoma against autologous melanoma cells and autologous normal lymphocytes. In addition, the B-lymphocyte reactivity with allogeneic melanoma cells was measured in patients with uveal melanoma and in healthy individuals. The significance of the results obtained was investigated by measuring the apparent B-lymphocyte reactivity with antigens which were unlikely to have been encountered previously by the individuals being tested. These were plant protoplasts from the tobacco plant as a cellular control, keyhole limpet haemocyanin as a purified protein control, and schistosome whole worm antigen as a complex mixture of foreign protein and carbohydrate.

We report here the findings of this investigation of the humoral immune response to uveal melanoma.

### Materials and methods

### *Preparation and transformation of human B-lymphocytes*

A single venous blood sample was obtained from 12 patients with uveal melanoma and from 5 healthy individuals. Two venous blood samples were taken from an additional patient with uveal melanoma for use in a reproducibility study.

Each blood sample (10–20 ml) was added to an equal volume of 2% foetal calf serum (FCS) in RPMI medium, layered over an equal volume of Ficoll–Paque, and centrifuged at 300 *g* for 15 min at room temperature. The cells at the fluid interface were harvested, washed in RPMI medium, and counted. They were then incubated at a concentration of $10^7$ cells ml$^{-1}$ for 1 h with EBV obtained from the supernatant of the B95-8 marmoset cell line (Miller & Lipman, 1973), then washed and aliquoted at $10^5$ cells/well in the central 60 wells of a 96-well Costar plate. Cyclosporin A, 1 $\mu$g ml$^{-1}$, was added to the 20% FCS medium to inhibit T-cell activity (Shevach, 1985) so that suppression of B-cell growth would be prevented (Bird *et al*., 1981). The transformed lymphocytes (transformants) were fed with fresh medium at weekly intervals. Supernatants were removed for assay in the interval spanning 10–21 days, during which time the cells continue to secrete large amounts of antibody.

### *Derivation of antigens*

*Relevant antigens* Blocks of uveal melanoma tissue (5 x 4 x 4 mm) were excised from 15 fresh tumours which were treated either by local surgical resection (Foulds, 1978) or by enucleation. Using the plunger of a disposable 10 ml syringe, the tumour tissue was passed through a stainless steel wire mesh into a 6 cm petri dish containing RPMI. A sample of the cell suspension was counted with a Neubauer haemocytometer and the integrity of the cells was determined by examining a parallel sample by phase contrast microscopy.

*Irrelevant antigens* Protoplasts were prepared from *Nicotiana plumbaginifolia* (tobacco plant) by the method of Sidorov and Maliga (1982). Keyhole limpet haemocyanin (KLH) was purchased from Sigma Chemicals, Poole, Dorset. Schistosome whole adult worm antigen was prepared by homogenisation of whole worms from *Schistosoma mansonii* in phosphate buffered saline followed by centrifugation at 500 *g* after 16 h incubation at room temperature, and was a gift from Dr Janet Jones, Department of Biochemistry, University of Glasgow.

### *Enzyme-linked immunosorbent assay (ELISA)*

The ELISA was similar to that described by Effros and associates (1985). Flat-bottomed, 96-well microtitre plates were rinsed briefly in warm distilled water. Aliquots of a cell suspension, consisting of $10^4$ cells/100 $\mu$g, were dispensed to all wells with the exception of row 1 of the plate. The plates were incubated in air at 37°C until they were completely dry and then wrapped in clingfilm and stored at 4°C until use. KLH and schistosome whole worm antigen were applied to the wells at concentrations of 10 $\mu$g 100 $\mu$l$^{-1}$ and 2 $\mu$g 100 $\mu$l$^{-1}$ respectively and incubated for 16 h at 4°C. The plates were then washed with PBS containing 0.05% Tween

Correspondence: B.E. Damato.
Received 23 February 1988; and in revised form, 3 May 1988.

and incubated with 100 $\mu$l transformant for 16 h at 4°C, After a further wash, the wells were incubated with 100 $\mu$l horseradish peroxidase-labelled rabbit anti-human IgG (H + L) (Dako) for 30 min at room temperature. This reagent detected IgM as well as antibodies by reaction with the constant region of the light chains. The wells were washed again and incubated with 100 $\mu$l orthophenylene diamine (0.4 mg ml$^{-1}$ in 0.01% $H_2O_2$). The reaction was arrested by the addition of 50 $\mu$l 4N $H_2SO_4$ and the light absorbance read at 492 nm using a Titretek Multiskan spectrophotometer. Six control wells on each plate contained 20% FCS instead of the transformant supernatant. Results greater than three standard deviations of the mean of the 6 control readings were considered to be positive.

*Reproducibility study*

A sample of venous blood was divided into two aliquots from which two preparations of lymphocytes were extracted. These were EBV-transformed separately and then assayed simultaneously on two different allogeneic uveal melanomas. One day after the first two transformations, EBV-transformation was performed on lymphocytes from a second blood sample from the same patient, and assayed against cells from the same two allogeneic uveal melanomas.

*Reactivity with autologous melanoma cells and lymphocytes*

The reaction of the peripheral B-cells of 5 patients with uveal melanoma was simultaneously tested against autologous tumour and B-lymphocytic cells.

*Reactivity with allogeneic melanoma cells and lymphocytes*

The reactions of peripheral B-lymphocytes of 5 patients with uveal melanoma and 5 healthy individuals were tested against allogeneic melanoma cells.

*Reactivity with irrelevant antigens*

The reactivities of peripheral B-lymphocytes of 2 patients with uveal melanoma and one healthy individual were tested against plant protoplasts, keyhole limpet haemocyanin and schistosome whole worm antigen. In each case, uveal melanoma was used as a positive control.

### Results

*Reproducibility study*

The reactivity of B-lymphocytes of patient JB with melanoma cells of patient MT was stronger than the reactivity with the melanoma cells of patient JS (Figure 1). The two lymphocyte preparations derived from the same blood sample showed good repeatability, that is, to within 10%. The lymphocyte preparation derived from the second blood sample showed good repeatability when tested against patient JS (i.e. 0–3%) and moderate repeatability when tested against patient MT (i.e. 13–18%).

*Reactivity with autologous melanoma cells and lymphocytes*

Three patients apparently reacted more strongly with the melanoma cells whereas 2 patients reacted more strongly with the lymphocytes (Figure 2).

*Reactivity of patients and healthy individuals with allogeneic melanoma cells*

The reactivity of the healthy individuals with the tumour cells was almost as strong as that seen in the patients with uveal melanoma (Figure 3).

*Reactivity with irrelevant antigens*

Figure 4 shows the reactivity of 3 individuals with irrelevant antigens consisting of plant protoplasts, keyhole limpet

**Figure 1** Reproducibility of measurement of ELISA reactivity of EBV transformed B-lymphocytes with allogeneic uveal melanoma cells. Two samples of lymphocytes prepared individually from the same venous blood sample of a patient (JB) with uveal melanoma were transformed separately (1 and 2). A third EBV transformation was performed on lymphocytes obtained one day later from the same patient (3). The reactivity of the three preparations of transformed B-lymphocytes with cells from two allogeneic uveal melanomas was measured by ELISA (JS and MT respectively). The histogram shows the percentage of transformants with optical density readings greater than the mean + 3 s.d. of 6 controls.

**Figure 2** ELISA reactivity of EBV transformed B-lymphocytes of patients with uveal melanoma with autologous tumour cells and lymphocytes. The results are obtained and displayed as in **Figure 1**. Three patients apparently reacted more strongly with the melanoma cells whereas two patients reacted more strongly with the lymphocytes.

**Figure 3** ELISA reactivity with allogeneic uveal melanoma cells of EBV transformed B-lymphocytes of patients with uveal melanoma and healthy individuals. The results are obtained and displayed as in **Figure 1**. The reactivity of the healthy individuals with the tumour cells was almost as strong as that seen in the patients with uveal melanoma.

**Figure 4** ELISA reactivity of EBV transformed B-lymphocytes of two patients with uveal melanoma (GL and ML) and one healthy individual (AR) with allogeneic uveal melanoma, protoplasts (P), keyhold limpet haemocyanin (KLH) and schistosome whole adult worm antigen (S). The results are obtained and displayed as in **Figure 1**. Positive reactivity was seen with all three antigens.

haemocyanin and adult schistosome whole worm antigen. Positive reactivity was seen with all 3 antigens.

### Discussion

Patients with uveal melanoma showed equal B-lymphocyte reactivity with autologous tumour cells and autologous lymphocytes. This suggested that the recognised antigens were not necessarily melanoma-associated. Other studies have apparently shown that patients with uveal melanoma and other types of malignancy have serum antibodies which are reactive with normal intracellular proteins (Malaty *et al.*, 1979).

The reactivity of the peripheral blood B-lymphocytes of healthy individuals with melanoma cells was very variable and similar to the allogeneic reactivity seen in patients with uveal melanoma. It is known that antibodies reactive with tumour-associated and normal tissue antigens occur in healthy individuals (Houghton *et al.*, 1980). Furthermore, monoclonal antibodies reactive with tumours and established autoantigens have previously been generated from unimmunised human individuals (Shoenfeld & Witz, 1986; Winger *et al.*, 1983). Such findings have given rise to speculation that these autoantibodies play a role in the regulation of the idiotypic network of both B- and T-lymphocytes (Schoenfeld & Witz, 1986).

Although the measurement of B-lymphocyte reactivity with melanoma cells from any individual tumour showed good to moderate reproducability, inconsistent results were obtained when the ELISA was simultaneously performed with different uveal melanomas. A similar observation was made previously (Damato *et al.*, 1986) and is likely to be due to inter-tumour antigenic heterogeneity, which is a well recognised phenomenon (Miller, 1982; Natali *et al.*, 1983; Heppner, 1984; Edwards, 1985).

The finding that B-lymphocyte-derived antibodies also reacted significantly with foreign antigens that were unlikely to have been encountered previously is not consistent with the hypothesis that auto-antibodies play useful roles *in vivo*. It may be argued that such results are an artefact of the ELISA, but such antibodies have been shown to react with intracellular material by other assays, such as immunofluorescence microscopy (Cote *et al.*, 1983) and immunoblotting (Cote *et al.*, 1986).

A possible explanation for the results of this study is that the antigens are wrongly identified by the antibodies that are secreted by the transformed B-lymphocytes. In human hybridoma technology, whether or not EBV transformation is employed, the great majority of immortalised lymphocytes secrete antibody of the IgM isotype (Steel *et al.*, 1977; Chan *et al.*, 1986; Cote *et al.*, 1986). This antibody, being multivalent, can react spuriously with various antigens in a 'multispecific' fashion because any low affinity interactions with such antigens will be amplified by local concentration effects (Ghosh & Campbell, 1986; Campbell *et al.*, 1987). The probability of this irrelevant type of non-specific reaction is increased when antigens have high epitope densities with a repeating structure offering a wide variety of regularly spaced epitopes. Such antigens include single stranded DNA, actin, myosin and vimentin (Ghosh & Campbell, 1986).

Multispecific antigen-antibody reactions, such as those seen in the present study, could account for the findings of previous investigations into the immunology of uveal melanoma. Serum IgM antibodies apparently reactive with intracellular proteins, such as actin, for example (Malaty *et al.*, 1979), could be directed against totally irrelevant antigens, such as bacterial and viral antigens. It is also conceivable that multispecific reactions with abundant cytoplasmic proteins have masked the presence of any antibodies directed against melanoma-associated membrane antigens. These considerations are also relevant in the production of rodent and human monoclonal antibodies to melanoma in that the apparent lack of specificity of an antibody could be due to multispecific reactivity rather than the expression of the target antigen in irrelevant cells.

Until such factors are taken into account, possibly by focusing attention on IgG antibodies reacting with autologous living cells, the existence of an *in vivo* humoral immune response to uveal melanoma will remain unproven.

Supported by the Scottish Hospital Endowments Research Trust (HERT 666).

### References

BIRD, G.A., McLACHLAN, S.M. & BRITTON, S. (1981). Cyclosporin A promotes spontaneous outgrowth *in vitro* of Epstein–Barr virus-induced B-cell lines. *Nature*, **289**, 300.

CAMPBELL, A.M., McCORMACK, M.A., ROSS, C.A. & LEAKE, R.E. (1986). Immunological analysis of the specificity of the autologous humoral response in breast cancer patients. *Br. J. Cancer*, **53**, 7.

CAMPBELL, A.M., WHITFORD, P. & LEAKE, R.E. (1987). Human monoclonal antibodies and monoclonal antibody multispecificity. *Br. J. Cancer*, **56**, 709.

CHAN, M.A., STEIN, L.D., DOSCH, H.-M. & SIGAL, N.H. (1986). Heterogeneity of EBV-transformable human B-lymphocyte populations. *J. Immunol.*, **136**, 106.

COTE, R.J., MORRISSEY, D.M., HOUGHTON, A.N., BEATTIE, E.J. JR., OETTGEN, H.F. & OLD, L.J. (1983). Generation of human monoclonal antibodies reactive with cellular antigens. *Proc. Natl Acad. Sci.* (*USA*), **80**, 2026.

COTE, R.J., MORRISSEY, D.M., HOUGHTON, A.N. & 4 others (1986). Specificity analysis of human monoclonal antibodies reactive with cell surface and intracellular antigens. *Proc. Natl Acad. Sci* (*USA*), **83**, 2959.

DAMATO, B.E., CAMPBELL, A.M., McGUIRE, B.J., LEE, W.R. & FOULDS, W.S. (1986). Monoclonal antibodies to human primary uveal melanomas demonstrate tumor heterogeneity. *Invest. Ophth. Vis. Sci.*, **27**, 1362.

EDWARDS, P.A.W. (1985). Heterogenous expression of cell-surface antigens in normal antibodies. *Br. J. Cancer.*, **51**, 14.

EFFROS, R.B., ZELLER, E., DILLARD, L. & WALFORD, R.L. (1985). Detection of antibodies to cell surface antigens by a simplified cellular elisa (CELISA). *Tissue Antigens*, **25**, 204.

FOULDS, W.S. (1973). Techniques for the local excision of choroidal melanomata. *Trans. Ophthal. Soc. UK.*, **93**, 343.

GHOSH, S. & CAMPBELL, A.M. (1986). Multispecific monoclonal antibodies. *Imm. Today*, **7**, 217.

HEPPNER, G.H. (1984). Tumor heterogeneity. *Cancer Res.*, **44**, 2259.

HOUGHTON, A.N., BROOKS, H., COTE, R.J., TAORMINA, M.C., OETTGEN, H.F. & OLD, L.J. (1983). Detection of cell surface and intracellular antigens by human monoclonal antibodies. Hybrid cell lines derived from lymphocytes of patients with malignant melanoma. *J. Exp. Med.*, **158**, 53.

HOUGHTON, A.N., TAORMINA, M.C., IKEDA, H., WATANABE, T., OETTGEN, H.F. & OLD, L.J. (1980). Serological survey of normal humans for natural antibody to cell surface antigens of melanoma. *Proc. Natl Acad. Sci.* (*USA*), **77**, 4260.

MALATY, A.H., RAHI, A.H. & GARNER, A. (1979). Ostensible anti-melanoma antibodies in patients with non-malignant eye disease. In *Immunology and Immunopathology of the Eye*, Silverstein, A.M. & O'Connor, G.R. (eds) p. 216. Masson: New York.

MILLER, F.R. (1982). Intratumor immunologic heterogeneity. *Cancer Met. Rev.*, **1**, 319.

MILLER, G. & LIPMAN, M. (1973). Release of infectious Epstein–Barr virus by transformed marmoset leukocytes. *Proc. Natl Acad. Sci,* (*USA*), **70**, 190.

NATALI, P.G., CAVALIERE, R., BIGOTTI, A. & 5 others (1983). Antigenic heterogeneity of surgically removed primary and autologous metastatic human melanoma lesions. *J. Immunol.*, **130**, 1462.

SCHOENFELD, Y. & WITZ1, I.P. (1986). Hybridomas from unimmunized individuals. *Immunol. Today*, **7**, 350.

SHEVACH, E.M. (1985). The effects of cyclosporin A on the immune system. *Ann. Rev. Immunol.*, **3**, 397.

SIDOROV, V.A. & MALIGA, P. (1982). Fusion-complementation analysis of auxotrophic and chlorophyll-deficient lines isolated in haploid *Nicotiana plumbaginifolia* protoplast cultures. *Mol. Gen. Genet.*, **186**, 328.

STEEL, C.M., PHILIPSON, J., ARTHUR, E., GARDINER, S.E., NEWTON, M.S., McINTOSH, R.V. (1977). Possibility of EB virus preferentially transforming a subpopulation of human B lymphocytes. *Nature*, **270**, 729.

STEINITZ, M., KOSKIMIES, S., KLEIN, G. & MAKELA, O. (1979). Establishment of specific antibody producing human lines by antigen preselection and Epstein–Barr virus (EBV)-transformation, *J. Clin. Lab. Immunol.*, **2**, 1.

WINGER, L., WINGER, C., SHASTRY, P., RUSSELL, A. & LONGENECKER, M. (1983). Efficient generation *in vitro*, from human peripheral blood cells, of monoclonal Epstein–Barr virus transformants producing specific antibody to a variety of antigens without prior deliberate immunization. *Proc. Natl Acad. Sci.* (*USA*), **80**, 4484.